# Decoding ‘Unnecessary Complexity’: A Law of Complexity and a Concept of Hidden Variation Behind “Missing Heritability” in Precision Medicine

**DOI:** 10.1007/s00239-021-10023-3

**Published:** 2021-08-02

**Authors:** Rama S. Singh

**Affiliations:** Department of Biology, and Origins Institute, McMaster University, 1280 Main Street West, Hamilton, ON L8S4K1 Canada

**Keywords:** Unnecessary complexity, Hidden variation, Missing heritability, Molecular evolution, Redundancy, Phenotypic complexity, Diluted heritability

## Abstract

The high hopes for the Human Genome Project and personalized medicine were not met because the relationship between genotypes and phenotypes turned out to be more complex than expected. In a previous study we laid the foundation of a theory of complexity and showed that because of the blind nature of evolution, and molecular and historical contingency, cells have accumulated *unnecessary complexity*, complexity beyond *what is necessary and sufficient to describe an organism*. Here we provide empirical evidence and show that unnecessary complexity has become integrated into the genome in the form of redundancy and is relevant to molecular evolution of phenotypic complexity. Unnecessary complexity creates uncertainty between molecular and phenotypic complexity, such that phenotypic complexity (*C*_P_) is higher than molecular complexity (*C*_M_), which is higher than DNA complexity (*C*_D_). The qualitative inequality in complexity is based on the following hierarchy: *C*_P_ > *C*_M_ > *C*_D_. This law-like relationship holds true for all complex traits, including complex diseases. We present a hypothesis of two types of variation, namely *open* and *closed* (hidden) systems, show that hidden variation provides a hitherto undiscovered “*third source*” of phenotypic variation, beside genotype and environment, and argue that “missing heritability” for some complex diseases is likely to be a case of “diluted heritability”. There is a need for radically new ways of thinking about the principles of genotype–phenotype relationship. Understanding how cells use hidden, *pathway variation* to respond to stress can shed light on why two individuals who share the same risk factors may not develop the same disease, or how cancer cells escape death.

## Introduction

In 2000, scientists unveiled the anatomy of the human genome, which is 3 billion nucleotides long from end to end. No other discovery in molecular biology—not the discovery of Mendel’s Laws, nor the discovery of DNA, nor the deciphering of the genetic code—came close to the Human Genome Project in arousing public jubilation in its implications for improved human health. It was as if Icarus had touched the Sun. But the Human Genome Project did not live up to its promise of better human health.

In terms of significance, the Human Genome Project has been compared to the Apollo program and the Manhattan Project in physics. However, biology is not physics. The Apollo program and the Manhattan Project were bottom-up, goal-oriented projects designed and executed to the end. They invented their “genotype” and produced their “phenotype”. In contrast, the HGP was a top-down project and represented the culmination of a long journey that began 100 years prior with the relation of genotypes to phenotypes to identify genes (Bateson and Mendel 1902). Ultimately, humans identified genes not by learning from phenotypes, but by crushing cells and tissues. This is a fact, not a criticism. Neil Armstrong could return from the moon because he remembered how he got there, and the bomb makers could diffuse the bomb because they remembered how they put it together. However, diseases turned out to be more complex than previously thought. A gene cannot be traced to a phenotype because the relationship between genotype and phenotype has turned out to be complex. Genes can, of course, be used from scratch to launch new projects and explore new territories. As an example, the success of messenger RNA-based vaccines against COVID-19 is a “Manhattan moment” of molecular biology and virology and shows the power of forward genomics.

Genomic medicine is defined as the “diagnosis, prognosis, prevention, and/or treatment of disease and disorders of the mind and body, using approaches informed or enabled by knowledge of the genome and the molecules it encode” (Buchanan et al. 2009). Genomic medicine promised individualized medicine, which, together with swiftly evolving technological change in rapid molecular diagnosis and data sharing, became known as personalized medicine. In this review, while the focus is on genomics, genomic medicine and personalized medicine will be used interchangeably.

The problem of precision medicine can be stated as follows: all individuals with the same risk factor for a given disease do not develop the disease. A notable example is the BRCA genes that cause breast and ovarian cancer in women. Individuals carrying either the BRCA1 or BRCA2 gene run a 50–85% lifetime risk of developing breast cancer and a 15–45% risk of ovarian cancer (Hamosh 2004). The existence of individuals carrying such major risk factors and not developing the disease could be due to a favorable environment, genotype–environment interaction, or even “protector genes”. As a second example, a Personal Genome Project Canada study using a sample of 56 whole genome sequences reported that 94% (53/56) of individuals carried at least one disease-associated allele (mean 3.3/individual); 25% (14/56) had a total of 19 alleles with other health implications, and an average of 3.9 genotypes associated with a risk of altered drug efficacy or reactions. Of the 19 health-implicated variants, six were “pathogenic or likely pathogenic”, but some caused no adverse symptoms in the carriers (Reuter et al. 2018), meaning risk factors do not have *fixed unalterable effects in their carriers.* The discovery that genes do not have *fixed effects* is a problem for precision medicine since it implies that there is no one-to-one relationship between genotype and phenotype. Investigations for causal genes for apparently highly heritable diseases have come up short, and the problem has been labeled as “missing heritability” (Manolio et al. 2009; Slatkin 2009; Eichler et al. 2010; Zuk et al. 2012). There is growing evidence of complex genetic interactions underlying most phenotypes (Sackton and Hartl 2016; Chow 2016; Mullis et al. 2018; Hou et al. 2018, 2019; Chen et al. 2016; Fournier and Schacherer 2017; Domingo et al. 2018), including Mendelian diseases in which the causal mutation is known (Cooper et al. 2013; Chow et al. 2016; Steinberg and Sebastiani 2012; Dorfman 2012; Cutting 2010).

With the goal of providing an evolutionary framework for the problem of the genotype–phenotype relationship and “missing heritability”, a previous work presented a theory of complexity, introduced the concept of unnecessary complexity (i.e., complexity beyond what is minimally necessary and sufficient to make an organism), and described how unnecessary complexity might underlie the lack of a one-to-one relationship between risk factors and diseases (Singh and Gupta 2020). Unnecessary complexity is not noise, not stochasticity, but rather an evolved property of living systems. It has been known by different names, including gene penetrance, expressivity, interaction, epistasis, modifiers, and background effect, but all point to the same thing, although these terms were more or less used as if they were limited to the genes and genotype, and the role of environment, G × E, and evolution were not of concern. Unnecessary complexity, in contrast, provides a comprehensive evolutionary framework for the complexity of gene interaction networks and underlying biochemical pathways.

To help take the concept of unnecessary complexity beyond the genome’s structural complexity and anchor it to the network of developmental pathways, this paper provides empirical evidence for unnecessary complexity and discusses its relevance to the nature of variation, cell evolvability, and phenotypic complexity. This review demonstrates (1) that the evolution of unnecessary complexity creates uncertainty between molecular and phenotypic complexity, which increases with evolutionary time, and states it the form of a law; (2) that besides molecular and historical contingency, gene and genome duplication is a persistent source of overlapping information and unnecessary complexity; (3) that unnecessary complexity resides as *alternate forms of biochemical pathways,* fixed or segregating among individuals just as nucleotide amino acid differences do; (4) that unnecessary complexity and the resulting *molecular redundancy* suggest the existence of shared genes and genotypes between traits and multiple paths from genotypes to phenotypes; and (5) that there are two types of variation—*open* variation, consisting of segregating alleles that respond to changes in the present environment, and *closed* variation, consisting of alternate pathways that are part of the organism’s stabilizing homeostatic systems and that respond only to sustained selection pressures. Unnecessary complexity or redundancy is not a setback to precision medicine; its consideration and understanding would revolutionize medical research and provide an evolutionary depth on redundant biochemical pathways as well as a basis for identifying gene networks and connecting genotypes to phenotypes. The Human Genome Project provided the *visible, physical genome*; what precision medicine needs is a second-generation Human Genome Project to unravel the *invisible, functional genome.*

## Theory of Complexity

A recent work elaborated on the nature of the relationship between genotype and phenotype, the relationship between chance-laden molecular complexity and the evolution of complex traits and laid the foundation of a theory of complexity and its relevance to precision medicine (Singh and Gupta 2020). Here we provide a brief summary of the theory.

Natural selection acts on phenotypes that do not have a one-to-one relationship with genotypes. All genes, good or bad, major or minor, reside in the same cell. Depending on the molecular contingency (i.e., the availability of particular sets of mutations, gene–gene interactions, cellular environment, and sampling effects), the same mutation will end up with different gene partners in different individuals or organisms. Given that organismal fitness depends on the function of all genes and how they interact, mutations with significant deleterious effects are eliminated. However, gene interaction and compensatory evolution lead to the persistence of many deleterious genes beyond their life expectancy. Thus, depending on the context, the same mutation can be deleterious in one individual, have a mild effect in a second, and have no effect in a third.

Context-dependent interactions or background effects arise from adaptive evolution or molecular contingency. The effect of molecular contingency (i.e., chance-driven molecular changes in conjunction with the blind nature of evolutionary processes) goes beyond the individual genes, includes role of environment, and creates genetic redundancy or *multiple molecular pathways to the same phenotype*. Contingency occurs at multiple time scale—physiological, gene expression, developmental, individual lifetime, etc. Over time, these pathways become more complex, interconnected, and hierarchically integrated. The theory of molecular complexity posits that it consists of two parts, *necessary* and *unnecessary* complexity, both of which are inseparable and increase over time (Singh and Gupta 2020). Complexity was defined as “*the number of gene–gene interactions and minimum biochemical path lengths necessary for a given molecular function, trait or organism*” and it was pointed out that “because of linkage, new mutations, new interactions, and, most importantly, the blind nature of the selection process, evolution necessarily leads to the creation of extra repetitious interactions of a roundabout nature, crisscrossing existing biochemical pathways and increasing the length of the molecular pathways” (Singh and Gupta 2020, p. 3); this was termed “unnecessary complexity”. The study suggested that necessary complexity, which comprises all aspects of an organism’s necessary genetic information, follows from DNA complexity (Cd) via intermediate molecules and their interaction, which is also context dependent. Unnecessary complexity, in contrast, is *evolutionary baggage* that is the result of molecular constraints, historical circumstances, and the blind nature of evolutionary forces, meaning selection makes use of what variation is available at the time with no regard to the future. “Unnecessary complexity” refers to how it arose, not how it is used. Unnecessary complexity has evolved, integrated and become part of the functional genome.

Unnecessary complexity has implications for evolutionary biology and molecular evolutionary theories. The blind nature of evolutionary forces makes evolution unpredictable, and unnecessary complexity exacerbates this unpredictability since genetic adaptation itself is highly unpredictable. The evolution of unnecessary molecular complexity and the resulting genetic redundancy create *uncertainty*, which creates an ever-increasing difference between DNA structural sequence complexity and phenotypic complexity. Notably, this has two effects. First, the invisible networks of molecular function that are not predictable from the structural properties of the genome would increase in complex organisms and create uncertainty, thereby making evolution even more unpredictable than that based on the probabilistic nature of population genetic processes. Second, and more importantly, no molecular theory of evolution based on the complexity of genome sequences or their various products (e.g., RNAs and proteins) alone can adequately explain the complexity (i.e., ontogeny, physiology, and function) of an organism.

## A Law of Complexity

The relationships between molecular and phenotypic complexity can be stated as a law based on a set of premises that can be summarized as follows from the principles of population genetics and evolution:

1. The phenotypic variance of a complex trait in any population at any time is greater than its genetic variance (*V*_P_ > *V*_G_).
2. The number of interacting gene partners for a new beneficial mutation increases as the mutation spreads through a population over time.
3. The complexity of biochemical pathways for a complex trait, in terms of path length and number of branches, increases over time.
4. The duplication of genetic information, for example through gene and genome duplication, combined with the blind nature of evolutionary processes and historical contingencies, leads to unnecessary complexity and redundancy.

**Fig. 1:**
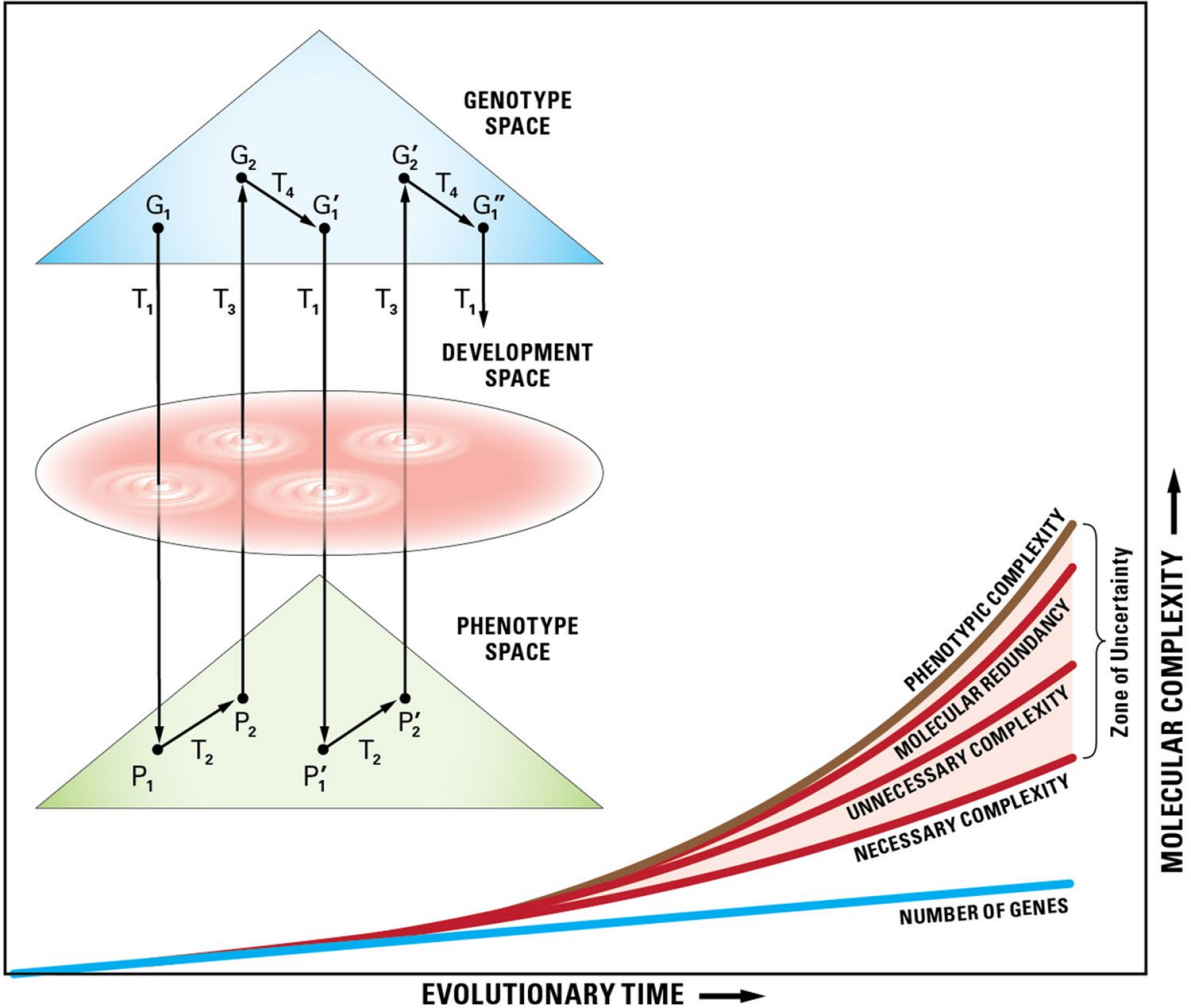
A diagrammatic representation of genotype–phenotype transformation from one generation to the next. G and P are the spaces of the genotypic and phenotypic description. G1, G′1, G2, and G′2 are genotypic descriptions at various time points within successive generations. P1, P′1, P2, and P′2 are phenotypic descriptions. T1 and T3 are laws of transformation from genotype to phenotype and back, respectively, during development. T2 is the law of population biology, and T4 relates to the laws of Mendel and Morgan regarding gamete formation. Necessary and unnecessary complexities, as well as molecular redundancy, are defined in the text. The graph lines are not intended to imply *monotonic increases* (after Lewontin 1972; Singh and Gupta 2020)

The law can be stated as follows: In evolution, molecular complexity (*C*_M_) increases over time (faster for unnecessary complexity than for necessary complexity) and the gap between necessary and unnecessary complexity constantly widens, resulting in phenotypic complexity (*C*_P_) being higher than molecular complexity (*C*_M_), which is higher than DNA complexity (C_D_). The qualitative inequality in complexity is based on the following hierarchy: *C*_P_ > *C*_M_ > *C*_D_ (Fig. 1).

The law of complexity implies two things. First, the higher up the evolutionary ladder an organism is, the larger the difference between molecular and phenotypic complexity. Second, no theory of evolution based on DNA sequence complexity at any given time alone would suffice to explain all aspects of an organism.

Unnecessary complexity can be verified by testing what the theory says, i.e., by showing that there are alternate biochemical pathways to a given complex phenotype beyond those defined by the segregating alleles. A second level of testing may involve checking the theory’s internal consistency as well as its agreement with other observations and predictions in the field, such as that: (1) diseases caused by de novo mutations, in general, would be less complex and less sexually dimorphic since the mutations would not have had time to evolve complex interactions, and (2) organisms living in a constant environment, for example, bacteria in a chemostat, would have less unnecessary complexity. There are several biological disciplines in which these ideas can be tested, e.g., phylogenetics, systems biological models, and experimental evolution.

## Sources of Unnecessary Complexity

There are two sources of unnecessary complexity that are embedded in how genes and genetic information arose through incremental increases in sequence complexity and segmented genes and how, over time, that information became duplicated in an overlapping manner through gene and genome duplication, giving rise to redundancy in the repertoire of genetic information. It would affect both protein coding and non-coding/regulatory information.

### Origin of Genetic Information: Segmented Genes, Disordered Proteins, and Binding Promiscuity

There is little information on how genetic information arose and how DNA pieces became linked together to make a chromosome or the genome. Recent studies have shown that a large proportion of proteins are partly or fully *disordered*, meaning they have no tertiary structure (Romero et al. 2006). A recent study of the human proteome showed a continuum of structure, with 37% of proteins fully folded with fixed tertiary structure, 58% containing folded regions with intrinsically disordered regions (IDRs), and 5% intrinsically disordered proteins (IDPs). Experimental studies have shown examples of important protein molecules that are partially or fully disordered in solution but retain their function (Kriwacki et al. 1996; Daughdrill et al. 1997). IDPs have been hailed as enablers of increased functional diversity in multicellular organisms (Romero et al. 2006; Uversky 2016a, b), involved in cellular signaling and regulation (Wright and Dyson 2015; Dyson 2016), associated with diseases (Babu et al. 2011), and an answer to the “missing heritability” of complex diseases (Tsang et al. 2020).

Besides playing an important role in gene regulation and diseases, the discovery of IDPs and IDRs provides an important window into the early stages of the evolution of genes and genetic information, as well as their effects on the evolving complexity of the genome. These molecules show how the evolution of genetic information in terms of sequence complexity and the number and size of genes would have been incremental, repetitive, and of a flexible binding nature. IDPs are said to be “characterized by an expanded sequence space, binding promiscuity, and conformational plasticity and polymorphism” (Uversky 2016a, b) before they become a fully foldable protein molecule. IDPs make an excellent model for the evolution of genes and genetic information. Most importantly, their binding promiscuity provides a model for the evolution of increasing gene interactions, functional interdependence, and trait complexity. The shared, deep, and overlapping ancestry of genes and their projection on the phenotype space provides a measure of the importance of ‘unnecessary complexity (Singh and Gupta 2020).

### Gene and Genome Duplication and Redundancy in Genetic Information

Genome size has evolved and increased via gene duplication (Ohno 2013), ranging in size from a single or a few nucleotides to a complete gene, a piece of a chromosome, a whole chromosome, or a whole genome, plus occasional contributions from viruses that include transposable elements. The average size of the duplication piece is expected to increase over time as the gene and genome increase in size. While gene duplication refers to the duplication of a piece of DNA, it also refers to the duplication of genetic information. New genes evolve via the functional differentiation of duplicated genes, but during the gene differentiation process, the new genes will likely retain some functional aspects of their “old selves” (i.e., earlier incarnations) and thus create overlapping layers of unnecessary complexity (i.e., “functional shadows of shadows”).

The cell is the building block of organisms and increasing complexity provides a molecular mechanism to record cellular experiences. Cellular (evolutionary) experiences are recorded not only in the *visible genome* via the sequence complexity of the DNA but also in the *invisible genome* via hidden variation in the form of biochemical pathways (both necessary and unnecessary) and the rules of vertical, long-distance, horizontal, and context-dependent interactions, as well as epigenetics.

## Evidence in Support of Unnecessary Complexity

Throughout the history of Mendelian genetics, there have been observations that did not fit the prevailing paradigm of the time and were set aside. While Barbara McClintock’s (1950) mobile elements produced spectacular and qualitatively distinct colored kernels in maize that fall into this class of observations, this review discusses a few less dramatic examples using invariable traits from population genetics.

### Evidence of Hidden Variation From Classic Selection Experiments in Population Genetics

In a seminal paper on “polygenic inheritance and natural selection”, Kenneth Mather (1943) introduced the concept of polygenes, in contrast to Mendelian oligogenes, and held that polygenes relate to quantitative, continuous variation, that species differences are polygenic, that “many genotypes may have the same phenotype”, and that “polygenic theory relates continuous phenotypic variation to discontinuous genotypic variation” (Mather 1943, p. 61). He believed that oligogenes and polygenes have different evolutionary potential and introduced the concept of *open or free vs. closed or hidden and potential variation*. Free variation is expressed in phenotypes and is selectable, while hidden variation is “hidden in the genotype under the cloak of phenotypic constancy”. Mather believed that “most variability in a population is potential” and that natural selection can release “undetectable potential to the detectable free state”. In Mather’s view, polygenes provide a mechanism for genomic storage, slow release, and “a genotypic flux to exist under the cloak of phenotypic stability” (Mather 1943, p. 63).

Organisms vary in quantitative traits such as shape and size. Over the years, countless laboratory selection experiments with model organisms have shown the operation of polygenic variation in terms of generally slow and incremental progress, and many animal and plant breeding programs are based on quantitative genetics theories (Falconer and Mackay 2009). It is important to point out that polygenes and polygenic variations were defined in terms of their individual effects only; in all other respects, they were Mendelian genes. Following are two examples that make polygenes something more than “genes of small effect”, which Mather alluded to as a “potential” and a “slow release” variation system.

Waddington (1953) reported the results of a selection experiment that he called “genetic assimilation of an acquired trait” using the crossveinless phenotype in *Drosophila melanogaster*. The crossveinless trait is rare in *D. melanogaster*, but Waddington increased its appearance by subjecting pupae to brief periods of high temperature, followed by artificial selection for high crossveinless individuals. He concluded that repeated cycles of heat treatment and artificial selection *released* the trait from *canalization* and made selectable genetic variation appear.

Rendel (1959) was able to increase the number of scute bristles in *D. melanogaster* by performing a sustained selection experiment aimed at showing that development was canalized around two, four, and possibly six bristles. He changed the population mean from two to five bristles and suggested that “to move from three bristles to five takes eight times the genetic change that it does to move from one bristle to three”, meaning *the higher the selectable phenotypic target, the larger the cumulative selection differential.* Like Waddington’s (1953), this experiment is an example of a non-linear relationship between genotype and phenotype that is subject to change and evolve.

All three above examples refer to *hidden variation*, a *stable phenotype,* and *non-linear selection response*, which are the properties of systems with interactions between genotypes and phenotypes (not between genes) and *non-linear dose–response relationships between genotypes and phenotypes.*

The University of Illinois’s long-term selection experiment for oil concentration in maize kernels provides, in my view, a genomic example of hidden variation being released and accounting for a smooth and sustained selection response. The selection experiment has been running for over a century, is still ongoing, and shows no sign of plateau (Laurie et al. 2004). A genomic comparison of selected lines showed that over 50 quantitative trait loci were involved, which accounted for only 50% of the genetic variance in oil content (Laurie et al. 2004). Maize is a cereal, not an oil crop. The selection response in oil concentration is likely to have involved the use of alternate pathways or the re-routing of biochemical pathways to convert carbohydrates into fat at a faster rate. Long-term selection experiments and selection response under stress such as the concept of “directed evolution” in bacteria (Hall 1991) may need re-evaluation in the light of ‘hidden variation’.

### Evidence of Hidden Variation From Genomics

The above examples provide indirect evidence of hidden variation. Direct evidence can come from genes and genomes, as demonstrated by the following two examples. There is growing evidence that phenotypes of genes are affected by their genetic backgrounds (Cooper et al. 2013; Chandler et al. 2014; Sackton and Hartl 2016; Chow 2016; Mullis et al. 2018; Hou et al. 2018; Chen et al. 2016; Fournier and Schacherer 2017; Domingo et al. 2018; Hou et al. 2019). Using two strains of yeast (*Saccharomyces cerevisiae*), Dowell et al. (2010) identified 1% of genes as conditionally essential, i.e., they were lethal in different genetic backgrounds. Hou et al. (2019) conducted a comprehensive genome mapping project that showed that conditional essential genes were present in multiple genomic regions: Over 20 genomic regions linked to seven conditional essential genes were identified (Hou et al. 2019). Interestingly, for a smaller set of genes, single modifiers were shown to be sufficient to rescue the phenotype; these were rare alleles (< 5%) of independent origin in natural populations (Hou et al. 2019). This study shows that molecular complexity is massive and multi-layered.

A study of restriction site–associated DNA sequence haplotypes, about 700 bp in length, in 50000 loci across the stickleback, a species of fish, genome showed fixation of haplotypes in geographically isolated freshwater pond populations that were identical by descent (Nelson and Cresko 2018). Sequence divergence of the same genomic region between freshwater and marine sticklebacks (measured as dxy/genetic distance) was tenfold higher than background levels. This is a remarkable demonstration of the fact that phenotypes of genes are affected by their genetic backgrounds. Even more remarkably, the standing genetic variation, which is the basis of adaptation between freshwater ponds, turned out to be about six million years old, nearly twice the genome-wide average. This study shows that molecular complexity of organisms is not only massive and multi-layered but also deep in terms of time. These results have implications for human health. While there is a tendency to assume that all human uniqueness evolved post human–chimpanzee splitting, it would be wrong to say that humans can be defined just by the total sum of their unique genes. It is more likely that what makes us human started unraveling long before the evolution of humans from chimpanzees. This view is supported by Goodman and Sterner (2010), who highlighted that “the close genetic correspondence of chimpanzees to humans and the relative shortness of our evolutionary separation suggest that most distinctive features of the modern human phenotype had already evolved during our ancestry with chimpanzees”. Unnecessary complexity is the mark of those old tracks.

## A Hypothesis of Hidden Variation: Polygenes, Pathways, and Norms of Reaction

This section brings the two ideas of unnecessary complexity and two types of variation (open vs. closed) together and proposes that unnecessary complexity would lead to the buildup of closed or hidden variation of the kind Mather (1943) had in mind. Mather introduced the concept of polygenes and open and closed variation partly based on the requirements of evolutionary theory and partly based on the discoveries at the time about the nature of variation in population and species. Phenotypic variation in shape and size appeared quantitative, polygenic—each with small effects as compared to non-heritable fluctuations—inexhaustible, and incremental in its response to selection. By “open variation” Mather meant segregating polymorphism, which can respond immediately to selection pressure, as in a changing environment. This is what Darwin’s theory required. Closed variation is different. Since Mather had no access to genomic details as we have today, he described closed variation in terms of segregating alleles locked in by selection and unable to respond immediately. He discussed the release of closed variation and the potential for response to sustained selection pressure. In Mather’s view, closed variation consisted of a balanced combination of polygenes, possibly a linkage block, resulting from stabilizing selection and phenotypic stability.

**Fig. 2:**
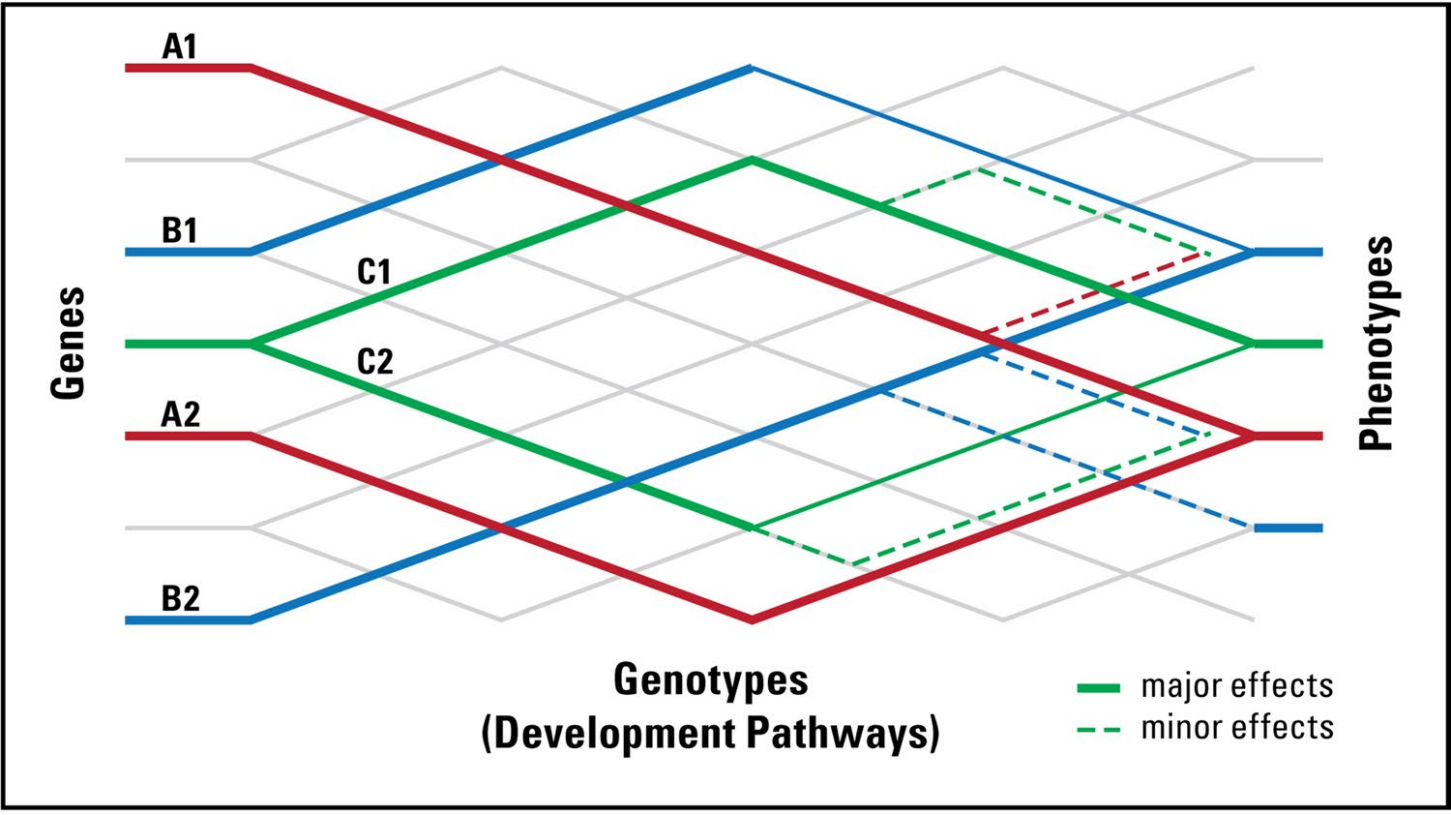
A diagrammatic representation of gene × gene interaction in biochemical pathways showing all possible interactions (whole grid in gray) superimposed by existing pathways (shown in color) depicting various kinds of effects between genes and traits: one gene affecting many traits, many genes affecting one trait, and a gene behaving as a major gene in case of one trait and a minor gene in case of another. Pathways become more complex over time. Pathways leading to a given trait can be treated as “alleles”, such as A1/A2, B1/B2, C1/C2, etc. (after Wright 1982)

In light of the concept of unnecessary complexity, closed or hidden variation can now be defined more clearly in terms of shared genotypes and multiple redundant developmental pathways varying in length, complexity, flux, threshold, and trait canalization (Fig. 2). Hidden variation in pathways can come from two sources (Table 1). First, there is no one-to-one relationship between segregating structural genomic variation and functional variation in biochemical pathways. All genetic elements, monomorphic or polymorphic, need not be actualized in the form of biochemical pathways at any given time. This would lead to the existence of alternate pathways in terms of path length, complexity, and flux. Second, more important to the idea of unnecessary complexity, alternate pathways can exist even in structurally monomorphic populations because *all* genes (regardless of whether they are monomorphic or polymorphic) do not engage in gene interaction at any given time. Variation can still occur in terms of alternate routes, thresholds, and rates of flux. Most of this variation is expected to be found in all individuals in a population but some can be found segregating between individuals. Long-term potentiality is what Mather (1943) meant by “closed variation”. Just as the two kinds of variation empower populations to evolve on different time scales, the two kinds of variation can act as a pair of evolutionary “genomic bifocals” and empower medical researchers to evaluate the complexity of diseases to determine whether they arise from short-term (open) or long-term (closed) variation. The open and closed variation systems can be viewed, respectively, as the chequing and the saving account of the cell. And just as the money in the chequing account is used first before drawing from the saving account, organisms make use of genetic polymorphisms to track the current environment, and use variation in the saving account for long-term use. Some complex diseases, barring those caused by de novo mutations, may owe their origins to changes in closed variation due to developmental perturbation (Singh 2004).

**Table 1:** Attributes of the open and closed system of genetic variation

Attributes	Open variation	Closed variation
Type of variation	Segregating DNA sequence variation, coding, non-coding	Alternate routes of biochemical pathways, varying in length, complexity, or rate of flux; shared genotypes between traits; redundant pathways behave like ‘fixed heterozygotes’ (see Fig. 2)
Source of variation	Mutation	Gene interaction, pathways alteration, selection, historical contingency
Fitness differences	Neutral or selectively maintained	Selectively maintained by stabilizing selection and homeostasis
Selection response	Responds to selection pressure from the present environment	Insensitive to short-term changes in the present environment; responds to medium-to-long-term sustained selection pressures
Rate of change	Generally slow, but it can be rapid if necessitated by the environment	Slow; time-lagged, punctuated change

**Fig. 3:**
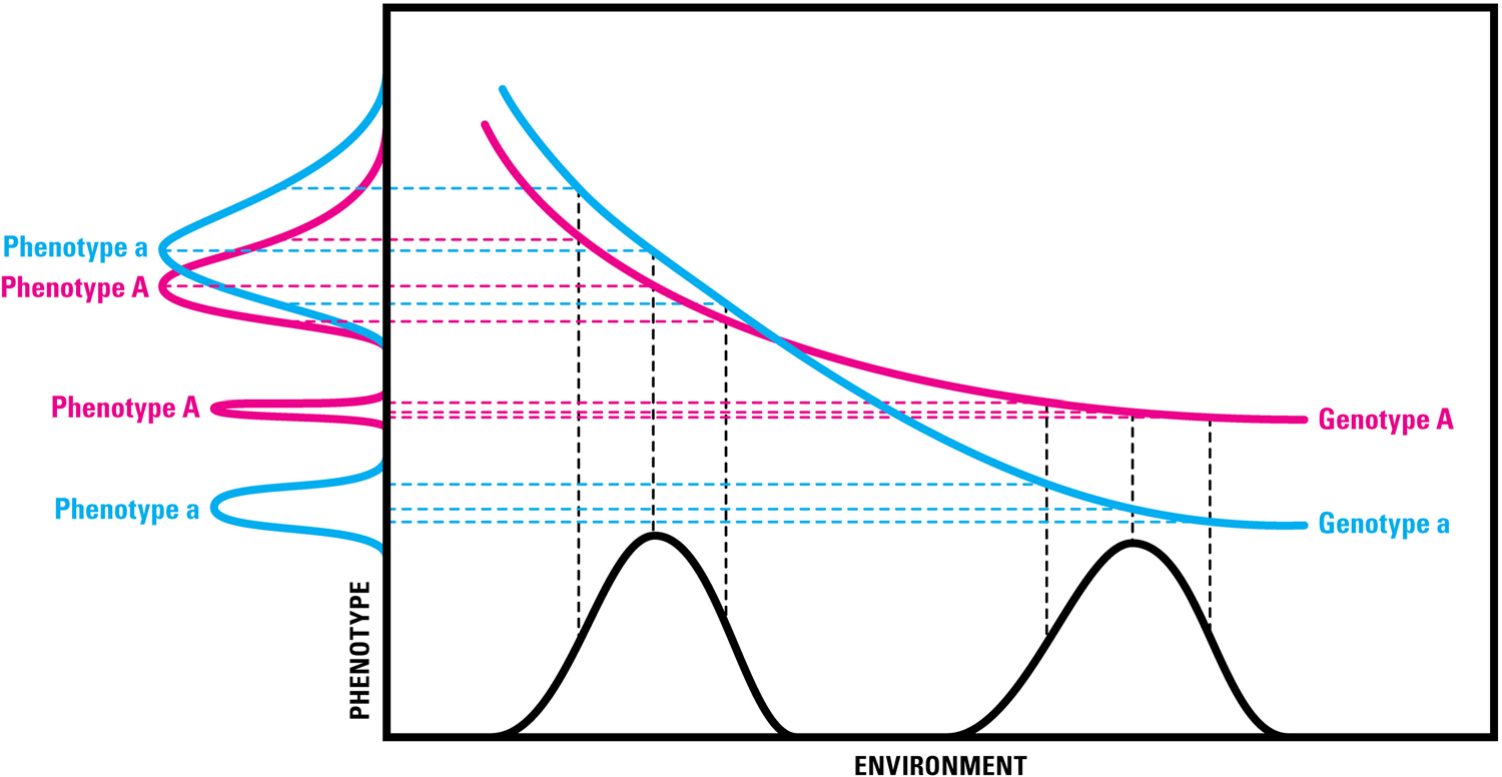
Norms of reaction showing relationship between genotypes and phenotypes in a range of environments (from Griffiths et al. 1996; with permission of the Author)

Hidden variation is exemplified by the presence of gene–gene interaction, or gene–environment interaction or norms of reaction (Fig. 3). Much literature still describes gene–environment (G x E) interaction as if it is a fixed and static property of the gene. However, it is anything but fixed. G x E interaction, as described by norms of reaction (Fig. 3), is dynamic, non-linear and unpredictable ((Lewontin 1974). There is growing evidence of high G x E interaction in complex diseases. As an example, a recent study of molecular liability and environmental exposures in schizophrenia showed that certain social factors had significant effects on the outcome of the disease, and, interestingly, the interaction effects were larger than the main effects. The study showed that sexual abuse, emotional abuse, emotional neglect, and bullying significantly affected the outcome of the disease (Guloksuz et al. 2019). Against a high heritability ranging from 73% for schizophrenia spectrum disorders to 79% for narrow schizophrenia diagnosis, polygenic risk scores are shown to explain only 7% of the variation on the scale of liability to schizophrenia. Main effects and interaction effects are interchangeable. Interaction effects in one environment can become part of the main effects in another environment. This is likely to be true of mental disorders as well (Singh et al. 2021).

## Pathway-Based Approaches Would Provide a Handle on Network Complexity

I will use four examples here to show how studies of variation and evolution at the level of pathway, rather than individual gene, are being used in a variety of ways to reduce the size of the network complexity and/or to understand the relationship between genotypes and phenotypes. First, just as Lewontin (1974) used the concept of norm of reaction, i.e., expression of genotypes over a range of environments, to show that G × E interaction was a dynamic and not a static process, *context-dependent* effects of genetic changes are being studied which are essentially norm of reaction of genes or genotypes as a function of other genes or genotypes (Costanzo et al. 2016; Kuzmin et al. 2018; Eguchi et al. 2019). Pathway-based consideration of genetic changes would reduce network complexity operationally and help connect genotypes to phenotypes. Second, simulation studies are being used to investigate the evolution of metabolic pathway function in the presence of mutation-selection -drift balance which can shed light on the evolution of rate limiting steps as well as on the role of compensatory vs. directional selection (Orlenko et al. 2016). Third, Noble and Hunter (2020) make the case for epigenomics-based quantitative (computational) physiological modeling to study relationship between genotypes and phenotypes. They rightly point out that low estimates of statistical genome-wide association between risk factors and disease are not necessarily indication of biological significance and small changes in a protein can have large fitness effect and vice versa. They show that physiological networks are robust to genetic changes and even if a key protein is missing, the rest of the network takes over. As an example, Hillenmeyer et al. (2008) showed that while complete deletion of most yeast genes (~ 80%) showed no obvious phenotypic effect in rich medium, under environmental stress conditions “97% of the gene deletion exhibited a measurable growth phenotype, suggesting that nearly all genes are essential for optimal growth in at least one condition”. (Hillenmeyer et al. 2008). This is the most direct evidence of condition-dependent fitness such that genomic redundancy provides a sink for deleterious mutations and have buffering effects on genetic change. Both genomic complexity- and physiology-based approaches are necessary to understand the relationship between genotypes and phenotypes. Finally, Bagchee-Clark et al. (2020) have shown that machine learning-based, pathway-extended gene expressions measurements can be successfully used to identify novel biomarkers that improve predictions of patient response to drugs in cancer therapy.

## The Problem of “Missing Heritability” in Precision Medicine

Successful implementation of precision medicine programs would require translational research from genotype to phenotype. Despite the success of genome-wide association studies (GWAS) in identifying common variants for complex traits, for the majority of complex diseases, < 10% of genetic variance is explained by common variants (Frazer et al. 2009). In many human Mendelian disorders in which the causal mutation is known, individuals carrying the mutation do not always develop the disease (Cooper et al. 2013; Chow et al. 2016; Steinberg and Sebastiani 2012; Dorfman 2012; Cutting 2010). The problem of “missing heritability”, i.e., that identified risk factors for a given disease do not fully account for the estimates of heritability based on family studies, has captured the concern of the precision medicine as well as genomic research community (Maher 2008; Manolio et al. 2009; Slatkin 2009; Eichler et al. 2010; Keller et al. 2012; Zuk et al. 2012; Nolte et al. 2017; Pallares 2019; Maroilley and Tarailo-Graovac 2019; López-Cortegano and Caballero 2019; Young 2019; Génin 2020, Douglas et al. 2020). Many suggestions have been offered regarding where to look for the “missing heritability”. These include pretty much everything: more in-depth studies assessing the role of structural variation such as copy number variation, deletion, duplication, inversions, numerous common variants of small effects, rare variants of large effects, parent of origin effects, microbiome, regulatory genes, the environment, imprinting, linkage and epistasis, microRNAs, complex genetic networks, context-dependent and non-additive effects, epigenetic inheritance, and gene interaction. Additionally, population demography, population structure, and recent population mixing and geneflow would add to population variation and haplotypes, making estimates of heritability spread over many loci. These are all important genetic phenomena and may account for missing heritability in varying degrees, but it is important to consider some cautions.

First, there is a fundamental difference between the heritability of quantitative traits such as human height and the heritability of complex diseases such as autism and diabetes which cause physiological disturbance and cellular stress. Height is positively selected over time and can be expected to be affected by many genes and by the environment. Autism, by virtue of syndrome heterogeneity, can be expected to vary between individuals and involve different genes, environments—developmental timing of particular environmental risk factors, and gene–environment interaction. Diabetes may turn out to be a classic Mendelian example of genotype–environment interaction. The heritability of complex diseases can be expected to vary from population to population.

Second, as the following example from *Drosophila* shows, a deeper study of hidden variation may be required to explain missing heritability. A study of three high-quality phenotypes (starvation resistance, chill coma recovery, and startle response) in ~ 200 inbred lines of *Drosophila melanogaster* showed that genotype (whole genome sequencing) and gene expression (deep RNA sequencing) in general had inconsistent and low predictability, both individually and jointly. The predictability improved when a gene ontology (GO) category was added as another level of information (Morgante et al. 2020). Since GO can involve different traits, these findings demonstrate the importance of shared genes between traits, as well as hidden variation.

Third, the problem of missing heritability may be overblown for a variety of reasons, and it may even be irrelevant. As Richard Lewontin (1974) noted, it is necessary to differentiate between the analysis of variance and the analysis of causes when diagnosing diseases. In relation to diseases, researchers need to know the number and nature of genes and their effects on the disease, not heritability. Heritability may be of intellectual interest, but it is of no use in most health situations as it has no predictive power over the “modifiability” of the trait (Feldman and Lewontin 1975). Heritability applies to population variance, not to population mean. As shown by the example of human height, heritability of a trait can be high within population and yet the trait’s mean values can be different between populations. For Mendelian diseases, genetic counseling based on family history is a widely used course of action. Individuals can respond to environmental intervention. What matters is mediation, not heritability.

Finally, in the case of predictive medicine using risk scores, while discovering rare variants of large effects would help, variants of small effect- common or rare- may not because small effect variants would be more susceptible to environmental perturbation and thus of less predictive value. Searching for missing heritability would be useful if a handful of major genes could be found to account for it. The results from the conditionally essential genes in yeast are somewhat encouraging. While most genes showed a complex underlying background, a few showed single modifiers that were rare and of independent origin. Over 80% of the naturally occurring variants in yeast were rare (< 5%), and the identified functional modifiers were also rare (< 5%). This raises the hope that, in some cases, rare variants may play an important role in rescuing “missing heritability” genes linked to the primary disease mutations (van Leeuwen et al. 2016).

## Hidden Variation—a Solution to the Problem of “Missing Heritability”?

In the above considerations regarding the search for genes to account for “missing heritability”, the focus is on segregating variation in the “visible genome”, i.e., segregating alleles in the genome. However, the missing variation may reside in the “invisible genome”, i.e., in the genetic variation in gene expression, locked up in the form of alternate biochemical pathways. It is quite likely that large GWAS may find enough polygenes collectively to account for the heritability of a complex disease. However, the polygene-based measures of heritability in large populations may not be the same as the measures of heritability based on family studies. This is because it cannot be assumed that the two measures are picking up the same type of genes. It is quite likely that the family-based high heritability is based on “developmental pathway–locked hidden variation” and is expressed like a unique set of genes in different genotypes. Both measures would be correct but different in their interpretation of the genetic causes.

Assuming that the genetic basis of the family-based heritability of complex diseases is the same as that measured from GWAS, it can be shown that the problem of “missing heritability” may really be a problem of “diluted heritability”. The underlying assumption for GWAS is that complex diseases are caused by the same set of genes in all families and populations. In this scenario, using a large sample size increases the signal-to-noise ratio and helps identify risk factors associated with the disease. In contrast, if the genetic basis of complex diseases is *not* the same in different families and populations, pooling samples from diverse sources would have the opposite effect of reducing the signal-to-noise ratio and thus reducing the chances of detecting risk factors.

As an example, let us assume, for the sake of simplicity, that autism is highly heritable within families and caused by different sets of five genes of varying effects in two unrelated families. We will call these families A and B, respectively. With five genes, the effect of some genes would still be substantial and measurable in an extensive GWAS performed within each family. Now suppose we pool the samples from the two families to increase the sample size and conduct a GWAS to identify risk factors. Pooling samples would not increase the frequency of a given risk factor; if anything the frequency will decrease and become rarer. In the pooled sample, even if members of the A family have the risk factors found in the B family, they would be unassociated with the disease. Similarly, members of the B family may have the risk factors found in the A family, but they would be unassociated with the disease. A GWAS would not benefit from a study of pooled samples, and it would lose all the signals or they would be diluted. The heritability detected within families is lost between families because the genes are different between families.

Now let us extend this example of heritability to many unrelated families that each have autistic children and different sets of unique genes. In the large, pooled sample, all individuals from a given family may have the risk factors found in other families, but they would be unassociated with the disease. A GWAS based on a pooled sample would not detect any stronger signal. In the pooled sample, the heritability would appear “lost”.

In real life, of course, all disease-causing genes in different families may not be different; some, if not most, are likely to be shared between families and populations, and some not. This would mean that in a pooled sample, the heritability of the complex disease would appear “diluted”, if not totally “lost”. The larger the number of unique genes (risk factors) per family that cause the disease, the larger the total number of genes involved in a pooled sample and the smaller the size of the individual gene effects. The scenario just described appears to fit the outcomes of GWAS with many complex diseases.

Barring effect of shared environments, the measurements of heritability in family studies may not be overestimated; it is likely that they are being underestimated in GWAS. This is because hidden (pathway) variation (*V*_PW_) would act as another source of genetic variation and while unrelated individuals would vary in this component, identical twins would share the same set of pathways. This may explain why twin-based estimates of heritability are always higher.

The new component of genetic variation (*V*_PW_) cannot be treated as part of the segregating sequence genetic variation component (*V*_G_); this is because pathways affect all genes, they would exercise their own *fixed* and *interactive* effects, nor can it be treated as part of the general environment because it is “in the DNA”. The prediction that the genetic basis of complex diseases between families and populations may not be the same is testable.

## Hidden Variation and Intangible Variance or “Irreducible Stochasticity”

An example of how the concept of hidden/pathway variation can provide resolution to a host of unexplained phenomena in biology is given by what can be called “irreducible stochasticity”. Stochasticity, by definition, cannot be removed completely but “irreducible stochasticity” refers to the fact that genetic variance in individuals and populations cannot be lowered to zero through inbreeding (Honegger and de Bivort 2018).

Stochastic variation in phenotypes are non-heritable effects that cannot be predicted from measurable variables. Honegger and de Bivort (2018) point out a classic case where a substantial amount of phenotypic variance remained even after holding genotype and environment as constant as possible. Gärtner (1990) showed that “30 years of inbreeding experiment on laboratory mice and rats in shared environments eliminated only 20–30% of observed variance in a number of phenotypes. The remaining 70–80% was referred as the *intangible variance*” (emphasis added). In terms of causes, the author speculated on the existence of a “*third component*” beside genotype and environment. Honegger and de Bivort (2018) cite other examples of identical genotypes producing dramatic differences in behavioral phenotypes and conclude that “stochastic individuality is a general phenomenon affecting essentially all behaviors in all species”. The authors conclude with the important role that stochastic variation plays in evolution.

The concept of hidden or pathway variation presented here can provide a mechanism to show that at least a portion of the stochastic variation can be explained to be as the result of hidden variation that is embedded in the structure of the developmental pathways affecting the trait. This portion of stochastic variance cannot be eliminated by inbreeding as this variation behaves like “*fixed heterozygosity*” making stochastic variation, strangely enough, appear as “heritable”. The hidden variation is the source of Gartner's “third component” (Gärtner 1990).

## The Way Forward: Decoding Unnecessary Complexity

The molecular complexity of diseases has put a damper on the high expectations for precision medicine, and the law of complexity stated above simply formalizes this problem. The paucity of high-frequency variants accounting for complex diseases is pushing the field toward large-scale population genomics and bioinformatics. A general understanding is emerging that biology is a science of variation and change, and this applies to disease and health as well. The role of unnecessary complexity, heterogeneity, and redundancy is a feature of all living things. One rule may not fit all complex diseases.

Precision medicine may need to follow a two-pronged approach: pushing ahead with investigating for “missing” causal disease genes and mounting disease/cell/tissue-based characterizations of biochemical pathways with the goal of developing cures. New approaches for capturing “missing heritability” genes of complex diseases would include (1) large-scale genomic studies of variant-specific phenotypes linked with computational bioinformatics, (2) in-depth studies of individuals from the same family with and without the disease, (3) comparative GWAS using the same variant from geographically isolated populations, (4) proteomics linked with molecular tagging and time-sequential cell-specific profiling, and possibly others. These approaches for predictive medicine are intended to capture mid- to high-frequency variants of high predictive value. Some serious thought would need to be given to the idea of differentiating “missing heritability” from “diluted heritability” and mounting testable predictions. This is a game-changing proposal as its confirmation could affect GWAS in significant ways.

A medicinal approach, in contrast, need not capture all the genes affecting a disease. Scientists only need to know one or two genes of large effect to be able to explore the biochemical pathways around those genes and develop a cure. Incidentally, unnecessary complexity, through pleiotropy, accounts for the side effects of medicines as well as tolerance to them. Humans would be worse off if it were not for the body’s homeostatic mechanism that minimizes the side effects of drugs. Unnecessary complexity contributes to those homeostatic mechanisms.

## Conclusions

The Human Genome Project has fallen short of its promise of personalized medicine (i.e., gene-driven development of healthcare in the form of gene therapy, gene-based drugs, or risk management) because the relationship between genotypes and phenotypes turned out to be more complex than initially expected. The concept of unnecessary complexity provides a conceptual breakthrough toward solving this problem. Unnecessary complexity translates into hidden variation in the form of biochemical pathway redundancy. The evolution of unnecessary complexity and resulting genetic redundancy create *molecular uncertainty*, which represents the ever-increasing gap between DNA structural complexity and organismic phenotypic complexity. This uncertainty increases as a function of complexity, meaning that more complex organisms are more removed from their genomic/DNA complexity, which has been stated as a law.

Success and scope of a theory depends on how many unsolved problems it can explain. Theory of complexity would have manifold effects in biology. A few examples follow: first, no molecular theory of evolution based on the complexity of genome sequences or their various products (e.g., RNAs and proteins) alone can adequately explain the complexity of an organism. Second, it would provide a solution to Muller’s problem with mutation load (Muller 1950) as redundancy would act as a sink for deleterious mutations and modulate their effect on fitness and disease (Johri ret a1. 2021; Singh and Gupta 2020). Third, it would provide a (non-neutral) solution to Lewontin’s dilemma of too much molecular variation (Lewontin 1974) as pathway redundancy would free individuals and populations from dependency on current mutations and would store genetic variation, accumulated as “experience” in the form of pre-adaptation, for future use (Delbrück 1949). Fourth, it would provide a solution to Haldane’s cost of evolution (Haldane 1957) as evolution would become free from being driven by individual genes. Fifth, it would provide a common meeting ground for population genetics and development and a solution to Goldschmidt and Gould’s problem of macroevolution (Goldschmidt 1940; Gould 1977; Gould and Eldredge 1977). Finally, most importantly, redundancy would confer freedom on individuals to negotiate their relationship with the environment.

To fully understand the complex relationship between genotypes and phenotypes, it is necessary to consider how the evolution of the gene and the genome came about. Genes and genomes are evolving, not static, entities. The early paucity of genes and the incremental increase in the size of the gene and genome in combination with the multi-faceted demand of the organism in the ever-changing environment would have led to *maximal use of genetic variation* through context-dependent gene–gene interaction, making one gene affect many traits and one trait be affected by many genes. The context- and interaction-led development of biochemical pathways or gene networks would have provided a mechanism for cellular memory to provide for the contradictory demands of the organisms, namely *organismic stability* within generations and *populational change* between generations.

The evolution of ever-increasing, unnecessary complexity over space (organisms) and time (evolution) makes a complete description of the molecular makeup of an organism theoretically inaccessible and, therefore, unknowable. Complexity and redundancy have a way of erasing the footprints of past molecular changes and make the “billion years of old experimentation” of the cell (Delbruck 1949) refractory to experimental investigation. On the plus side, understanding how the body cells make use of running and saving, or open and closed, accounts of mutations has the potential to shed light on the molecular basis of complex traits including complex diseases and mental disorders.

The Human Genome Project unraveled the *visible* (structural) genome, but a similar or greater effort will be required to decode the complex biochemical pathways, i.e., the *invisible* (functional) genome/pathways, that connect genotypes to phenotypes (Fig. 3). The journey from genes to phenotypes may not take another 100 years, but it promises to be challenging.

## Abbreviations

HGP: Human genome project
BRCA: Breast cancer
IDPs: Intrinsically disordered proteins
IDRS: Intrinsically disordered regions
GWAS: Genome wide association studies

## References

[CR1] Babu MM, van der Lee R, de Groot NS, Gsponer J (2011). Intrinsically disordered proteins: regulation and disease. Curr Opin Struct Biol.

[CR2] Bagchee-Clark AJ, Mucaki EJ, Whitehead T, Rogan PK (2020). Pathway-extended gene expression signatures integrate novel biomarkers that improve predictions of patient responses to kinase inhibitors. MedComm.

[CR3] Bateson W, Mendel G (1902). Mendel’s principles of heredity: a defence, with a translation of Mendel’s original papers on hybridisation.

[CR4] Buchanan JA, Carson AR, Chitayat D, Malkin D, Meyn MS, Ray PN, Shuman C, Weksberg R, Scherer SW (2009). The cycle of genome-directed medicine. Genome Med.

[CR76] Chandler CH, Chari S, Tack D, Dworkin I (2014). Causes and consequences of genetic background effects illuminated by integrative genomic analysis. Genetics.

[CR5] Chen R, Shi L, Hakenberg J (2016). Analysis of 589,306 genomes identifies individuals resilient to severe Mendelian childhood diseases. Nat Biotechnol.

[CR6] Chow CY (2016). Bringing genetic background into focus. Nat Rev Genet.

[CR7] Chow CY, Kelsey KJP, Wolfner MF, Clark AG (2016). Candidate genetic modifiers of retinitis pigmentosa identified by exploiting natural variation in Drosophila. Hum Mol Genet.

[CR8] Cooper DN, Krawczak M, Polychronakos C (2013). Where genotype is not predictive of phenotype: towards an understanding of the molecular basis of reduced penetrance in human inherited disease. Hum Genet.

[CR9] Costanzo M, VanderSluis B, Koch EN (2016). A global genetic interaction network maps a wiring diagram of cellular function. Science.

[CR10] Cutting GR (2010). Modifier genes in Mendelian disorders: the example of cystic fibrosis: modifiers of cystic fibrosis. Ann N Y Acad Sci.

[CR11] Daughdrill GW, Chadsey MS, Karlinsey JE (1997). The C-terminal half of the anti-sigma factor, FlgM, becomes structured when bound to its target, σ28. Nat Struct Mol Biol.

[CR12] Delbrück M (1949). A physicist looks at biology. Trans Conn Acad Arts Sci.

[CR13] Domingo J, Diss G, Lehner B (2018). Pairwise and higher-order genetic interactions during the evolution of a tRNA. Nature.

[CR14] Dorfman R (2012). Modifier gene studies to identify new therapeutic targets in cystic fibrosis. Curr Pharm Des.

[CR15] Douglas GM, Bielawski JP, Langille MGI (2020). Re-evaluating the relationship between missing heritability and the microbiome. Microbiome.

[CR16] Dowell RD, Ryan O, Jansen A (2010). Genotype to phenotype: a complex problem. Science.

[CR17] Dyson HJ (2016). Making sense of intrinsically disordered proteins. Biophys J.

[CR18] Eguchi Y, Bilolikar G, Geiler-Samerotte K (2019). Why and how to study genetic changes with context-dependent effects. Curr Opin Genet Dev.

[CR19] Eichler EE, Flint J, Gibson G (2010). Missing heritability and strategies for finding the underlying causes of complex disease. Nat Rev Genet.

[CR20] Falconer DS, Mackay T (2009). Introduction to quantitative genetics.

[CR21] Feldman M, Lewontin R (1975). The heritability hang-up. Science.

[CR22] Fournier T, Schacherer J (2017). Genetic backgrounds and hidden trait complexity in natural populations. Curr Opin Genet Dev.

[CR23] Frazer KA, Murray SS, Schork NJ, Topol EJ (2009). Human genetic variation and its contribution to complex traits. Nat Rev Genet.

[CR24] Gärtner K (1990). A third component causing random variability beside environment and genotype. A reason for the limited success of a 30 year long effort to standardize laboratory animals?. Lab Anim.

[CR25] Génin E (2020). Missing heritability of complex diseases: case solved?. Hum Genet.

[CR26] Goldschmidt R (1940). The material basis of evolution.

[CR27] Goodman M, Sterner KN (2010). Phylogenomic evidence of adaptive evolution in the ancestry of humans. Proc Natl Acad Sci USA.

[CR28] Gould SJ (1977). The return of hopeful monsters. Nat Hist.

[CR29] Gould SJ, Eldredge N (1977). Punctuated equilibria: the tempo and mode of evolution reconsidered. Paleobiology.

[CR30] Guloksuz S, Pries L, Delespaul P (2019). Examining the independent and joint effects of molecular genetic liability and environmental exposures in schizophrenia: results from the EUGEI study. World Psychiatry.

[CR31] Haldane JBS (1957). The cost of natural selection. J Genet.

[CR32] Hall BG (1991). Adaptive evolution that requires multiple spontaneous mutations: Mutations involving base substitutions. Proc Natl Acad Sci.

[CR33] Hamosh A (2004). Online Mendelian Inheritance in Man (OMIM), a knowledgebase of human genes and genetic disorders. Nucleic Acids Res.

[CR75] Hillenmeyer ME, Fung E, Wildenhain J, Pierce SE, Hoon S, Lee W, Proctor M, St.Onge RP, Tyers M, Koller D, Altman RB, Davis RW, Nislow C, Giaever G (2008). The chemical genomic portrait of yeast: uncovering a phenotype for all genes. Science.

[CR34] Honegger K, de Bivort B (2018). Stochasticity, individuality and behavior. Curr.

[CR35] Hou J, van Leeuwen J, Andrews BJ, Boone C (2018). Genetic network complexity shapes background-dependent phenotypic expression. Trends Genet.

[CR36] Hou J, Tan G, Fink GR (2019). Complex modifier landscape underlying genetic background effects. Proc Natl Acad Sci USA.

[CR38] Johri P, Charlesworth B, Howell EK, Lynch M, Jensen JD (2021). Revisiting the notion of deleterious sweeps. Genetics.

[CR39] Keller MF, Saad M, Bras J (2012). Using genome-wide complex trait analysis to quantify “missing heritability” in Parkinson’s disease. Hum Mol Genet.

[CR40] Kriwacki RW, Ludger H, Tennant L, Tennant L, Reed SI, Wright PE (1996). Structural studies of p2lWa1CiPl/Sdil in the free and Cdk2-bound state: conformational disorder mediates binding diversity. Proc Natl Acad Sci USA.

[CR41] Kuzmin E, VanderSluis B, Wang W (2018). Systematic analysis of complex genetic interactions. Science.

[CR42] Laurie CC, Chasalow SD, LeDeaux JR, McCarroll R, Bush D, Hauge B, Lai C, Clark D, Rocheford TR, Dudley JW (2004). The genetic architecture of response to long-term artificial selection for oil concentration in the maize kernel. Genetics.

[CR43] Lewontin RC (1974). Annotation: the analysis of variance and the analysis of causes. Am J Hum Genet.

[CR44] López-Cortegano E, Caballero A (2019). Inferring the nature of missing heritability in human traits using data from the GWAS catalog. Genetics.

[CR45] Maher B (2008). Personal genomes: the case of the missing heritability. Nature.

[CR46] Manolio TA, Collins FS, Cox NJ (2009). Finding the missing heritability of complex diseases. Nature.

[CR47] Maroilley T, Tarailo-Graovac M (2019). Uncovering missing heritability in rare diseases. Genes.

[CR48] Mather K (1943). Polygenic inheritance and natural selection. Biol Rev.

[CR49] McClintock B (1950). The origin and behavior of mutable loci in maize. Proc Natl Acad Sci USA.

[CR50] Morgante F, Huang W, Sørensen P, Maltecca C, Mackay TFC (2020). Leveraging multiple layers of data to predict drosophila complex traits. Genes Genom Genet.

[CR51] Mullis MN, Matsui T, Schell R, Foree R, Ehrenreich IM (2018). The complex underpinnings of genetic background effects. Nat Commun.

[CR52] Nelson TC, Cresko WA (2018). Ancient genomic variation underlies repeated ecological adaptation in young stickleback populations. Evol Lett.

[CR53] Nolte IM, van der Most PJ, Alizadeh BZ (2017). Missing heritability: is the gap closing? An analysis of 32 complex traits in the Lifelines Cohort Study. Eur J Hum Genet.

[CR54] Ohno S (2013). Evolution by gene duplication.

[CR55] Orlenko A, Teufel AI, Chi PB, Liberles DA (2016). Selection on metabolic pathway function in the presence of mutation-selection-drift balance leads to rate-limiting steps that are not evolutionarily stable. Biol Direct.

[CR56] Pallares LF (2019). Searching for solutions to the missing heritability problem. Elife.

[CR57] Rendel JM (1959). Canalization of the scute phenotype of drosophila. Evolution.

[CR58] Reuter MS, Walker S, Thiruvahindrapuram B (2018). The personal genome project Canada: findings from whole genome sequences of the inaugural 56 participants. Can Med Assoc J.

[CR59] Romero PR, Zaidi S, Fang YY, Uversky VN, Radivojac P, Oldfield CJ, Cortese MS, Sickmeier M, LeGall T, Obradovic Z, Dunker AK (2006). Alternative splicing in concert with protein intrinsic disorder enables increased functional diversity in multicellular organisms. Proc Natl Acad Sci USA.

[CR60] Sackton TB, Hartl DL (2016). Genotypic context and epistasis in individuals and populations. Cell.

[CR61] Singh RS (2004). Genomic bi-focals and a panoramic view of evolution. Trends Ecol Evol.

[CR62] Singh RS, Gupta BP (2020). Genes and genomes and unnecessary complexity in precision medicine. NPJ Genom Med.

[CR74] Singh RS, Singh KK, Singh SM (2021). Origin of sex-biased mental disorders: an evolutionary perspective.. J Mol Evol.

[CR63] Slatkin M (2009). Epigenetic inheritance and the missing heritability problem. Genetics.

[CR64] Steinberg MH, Sebastiani P (2012). Genetic modifiers of sickle cell disease. Am J Hematol.

[CR65] Tsang B, Pritišanac I, Scherer SW, Moses AM, Forman-Kay JD (2020). Phase separation as a missing mechanism for interpretation of disease mutations. Cell.

[CR66] Uversky VN (2016). Dancing protein clouds: the strange biology and chaotic physics of intrinsically disordered proteins. J Biol Chem.

[CR67] Uversky VN (2016). Paradoxes and wonders of intrinsic disorder: complexity of simplicity. Intrinsically Disord Proteins.

[CR68] van Leeuwen J, Pons C, Mellor JC (2016). Exploring genetic suppression interactions on a global scale. Science.

[CR69] Waddington CH (1953). Genetic assimilation of an acquired character. Evolution.

[CR71] Wright PE, Dyson HJ (2015). Intrinsically disordered proteins in cellular signalling and regulation. Nat Rev Mol Cell Biol.

[CR72] Young AI (2019). Solving the missing heritability problem. PLoS Genet.

[CR73] Zuk O, Hechter E, Sunyaev SR, Lander ES (2012). The mystery of missing heritability: genetic interactions create phantom heritability. Proc Natl Acad Sci USA.

